# Staphylococcus aureus from Atopic Dermatitis Patients: Its Genetic Structure and Susceptibility to Phototreatment

**DOI:** 10.1128/spectrum.04598-22

**Published:** 2023-05-04

**Authors:** Patrycja Ogonowska, Klaudia Szymczak, Joanna Empel, Małgorzata Urbaś, Agata Woźniak-Pawlikowska, Wioletta Barańska-Rybak, Dariusz Świetlik, Joanna Nakonieczna

**Affiliations:** a Intercollegiate Faculty of Biotechnology, University of Gdansk and Medical University of Gdansk, Gdańsk, Poland; b Department of Epidemiology and Clinical Microbiology, National Medicines Institute, Warsaw, Poland; c Department of Dermatology, Venereology and Allergology, Medical University of Gdańsk, Gdańsk, Poland; d Division of Biostatistics and Neural Networks, Medical University of Gdańsk, Gdańsk, Poland; Institut Necker Enfants Malades

**Keywords:** atopic eczema, MRSA/MSSA, *spa* type, clonal complex, superantigen (SAg), staphylococcal enterotoxin, rose bengal, photodynamic inactivation, visible light

## Abstract

We characterized the population of Staphylococcus aureus from patients with atopic dermatitis (AD) in terms of (i) genetic diversity, (ii) presence and functionality of genes encoding important virulence factors: staphylococcal enterotoxins (*sea*, *seb*, *sec*, *sed*), toxic shock syndrome 1 toxin (*tsst*-1), and Panton-Valentine leukocidin (*lukS*/*lukF*-*PV*) by spa typing, PCR, drug resistance profile determination, and Western blot. We then subjected the studied population of S. aureus to photoinactivation based on a light-activated compound called rose bengal (RB) to verify photoinactivation as an approach to effectively kill toxin-producing S. aureus. We have obtained 43 different *spa* types that can be grouped into 12 clusters, indicating for the first-time clonal complex (CC) 7 as the most widespread. A total of 65% of the tested isolates had at least one gene encoding the tested virulence factor, but their distribution differed between the group of children and adults, and between patients with AD and the control group without atopy. We detected a 3.5% frequency of methicillin-resistant strains (MRSA) and no other multidrug resistance. Despite genetic diversity and production of various toxins, all isolates tested were effectively photoinactivated (bacterial cell viability reduction ≥ 3 log_10_ units) under safe conditions for the human keratinocyte cell line, which indicates that photoinactivation can be a good option in skin decolonization.

**IMPORTANCE** Staphylococcus aureus massively colonizes the skin of patients with atopic dermatitis (AD). It is worth noting that the frequency of detection of multidrug-resistant S. aureus (MRSA) in AD patients is higher than the healthy population, which makes treatment much more difficult. Information about the specific genetic background of S. aureus accompanying and/or causing exacerbations of AD is of great importance from the point of view of epidemiological investigations and the development of possible treatment options.

## INTRODUCTION

Atopic dermatitis (AD) is a chronic inflammatory disease with a complex pathophysiological mechanism, including skin barrier defects, exposure to allergens, and significant dysbiosis of the skin microbiome. Mutations in filaggrin have been shown to be associated with an atopic phenotype among people, as well as lowered levels of skin antimicrobial peptides, e.g., cathelicidin and β-defensin. Such conditions promote colonization of skin with Staphylococcus aureus. This bacterium colonizes lesions of AD skin, and its presence has been demonstrated to correlate with disease severity (1–5). It is commonly accepted that S. aureus plays a role in AD; however, its role in promoting and/or enabling the disease has not been elucidated. Some authors hypothesize that skin inflammation is exacerbated by S. aureus penetration below the epidermis, where bacterial cells increase protease activity in keratinocytes, disrupting the skin barrier (6).

S. aureus exacerbates AD symptoms by producing several enterotoxins that possess superantigenic properties, overstimulating Th lymphocytes (7). Patients colonized with superantigen (SAg)-producing S. aureus produce IgE antibodies directed against SAgs, and a strong correlation with disease severity is observed in those patients (8). Even more interestingly, SAgs induce corticosteroid resistance, thus seriously complicating treatment and response to AD therapy (9). In the case of mild AD, optimal skin care, such as the application of emollients and skin hydration, is used as basic treatment. With increasing disease severity, topical corticosteroids (TCSs) and/or calcineurin inhibitors are applied. However, there is limitation with the use of TCSs, particularly on delicate skin (e.g., face and genital area). A separate treatment issue concerns children, in whom only mild steroid preparations should be used (10). Topical calcineurin inhibitors (TCIs) have been proven to be effective in AD, as they have antiinflammatory potency, and the risk of skin atrophy is low (11); however, long-term safety data are not available. pimecrolimus cream is registered for use in children from the age of 2, as well as 0.03% tacrolimus ointment, both constituting a safe option for children. Children are treated with so-called proactive therapy twice per week after controlling acute inflammation, which prevents disease relapse and progression.

Due to heavy S. aureus colonization of the affected but also nonaffected skin of AD patients, secondary skin infections such as impetigo, folliculitis, and furunculosis are quite common. The main antibiotics used in their treatment are fusidic acid and mupirocin. Unfortunately, long-term therapy with antibiotics selects for increasing resistance (12). In terms of the severe form of the disease, systemic treatment with antimicrobials, corticosteroids, cyclosporine, or antihistamines could be applied. However, a problem exists; when the treatment is discontinued, disease relapse is often observed. Moreover, there are serious side effects documented with systemic corticosteroid or cyclosporine treatments; therefore, only a short course of therapy may be beneficial for AD patients (13). In recent years, a series of targeted AD therapies based on multiple agents have demonstrated efficacy, among which only dupilumab is currently approved for adults with AD (14). A well-established second-line treatment is phototherapy with ultraviolet radiation A (UVA) and ultraviolet radiation B (UVB). In AD patients, the following options are available: broad-band UVB (280 to 320 nm), narrow-band UVB (311 to 313 nm), UVA (340 to 400 nm), and PUVA (UVA together with photosensitizing compounds – psoralens). These options are limited to children aged 12 years and older, as long-term effects of UV-based therapies are still not known. It is important to provide a safe alternative that could overcome the problem of resistance to antibiotics and could be based on safer longer wavelength lights and thus potentially applied in children.

Antimicrobial photodynamic inactivation (aPDI) constitutes a viable option for antistaphylococcal treatment that could be applied topically, with a local selective delivery of light of longer and thus safer nonmutagenic wavelengths (15, 16). aPDI is based on the use of a nontoxic small molecule compound called a photosensitizer (PS) and light from the visible spectrum that is absorbed by the PS. Such a PS in the presence of oxygen generates several types of reactive oxygen species (ROS) that are responsible for the toxic effect toward microbial cells. The advantage of aPDI is the fact that several consecutive treatments do not lead to resistance to aPDI, and moreover, various isolates can be effectively eradicated independent of their antibiotic resistance profile.

The importance of skin colonization with S. aureus, postulated as an element of AD pathogenesis, underlines antistaphylococcal treatment as a viable approach to improve the conditions of inflamed skin of AD patients. There is an ongoing debate concerning the genetic diversity of AD-derived strains, including those producing enterotoxins. Specifically, it has been postulated that S. aureus skin isolates produce elevated amounts of staphylococcal enterotoxins (SEs). Some authors have shown that certain SEs are associated with particular clonal lineages (reviewed by Ogonowska et al. [17]).

The aim of this work was to assess the distribution and genetic variation of S. aureus originating from AD patients and to verify the hypothesis that regardless of the genetic background, skin isolates can be effectively eradicated by photoinactivation. Although some data on the genetic diversity of AD-derived S. aureus exist (17), research on S. aureus, from both its primary ecological niche, anterior nares, and inflamed skin of the same AD patient are scarce. Therefore, we aimed to evaluate the clonal relatedness of S. aureus isolates from nasal carriage and skin lesions of AD patients, both children and adults. We were interested in the distribution of genes coding for selected SAg, staphylococcal enterotoxins SEA-D and TSST-1, and their functionality in *in vitro* culture conditions. In the studied population, we also analyzed the presence of the genes encoding Panton-Valentine leucocidin (PVL). Furthermore, we investigated *in vitro* photoinactivation of those AD-derived isolates.

## RESULTS

### The population of AD isolates.

**FIG 1:**
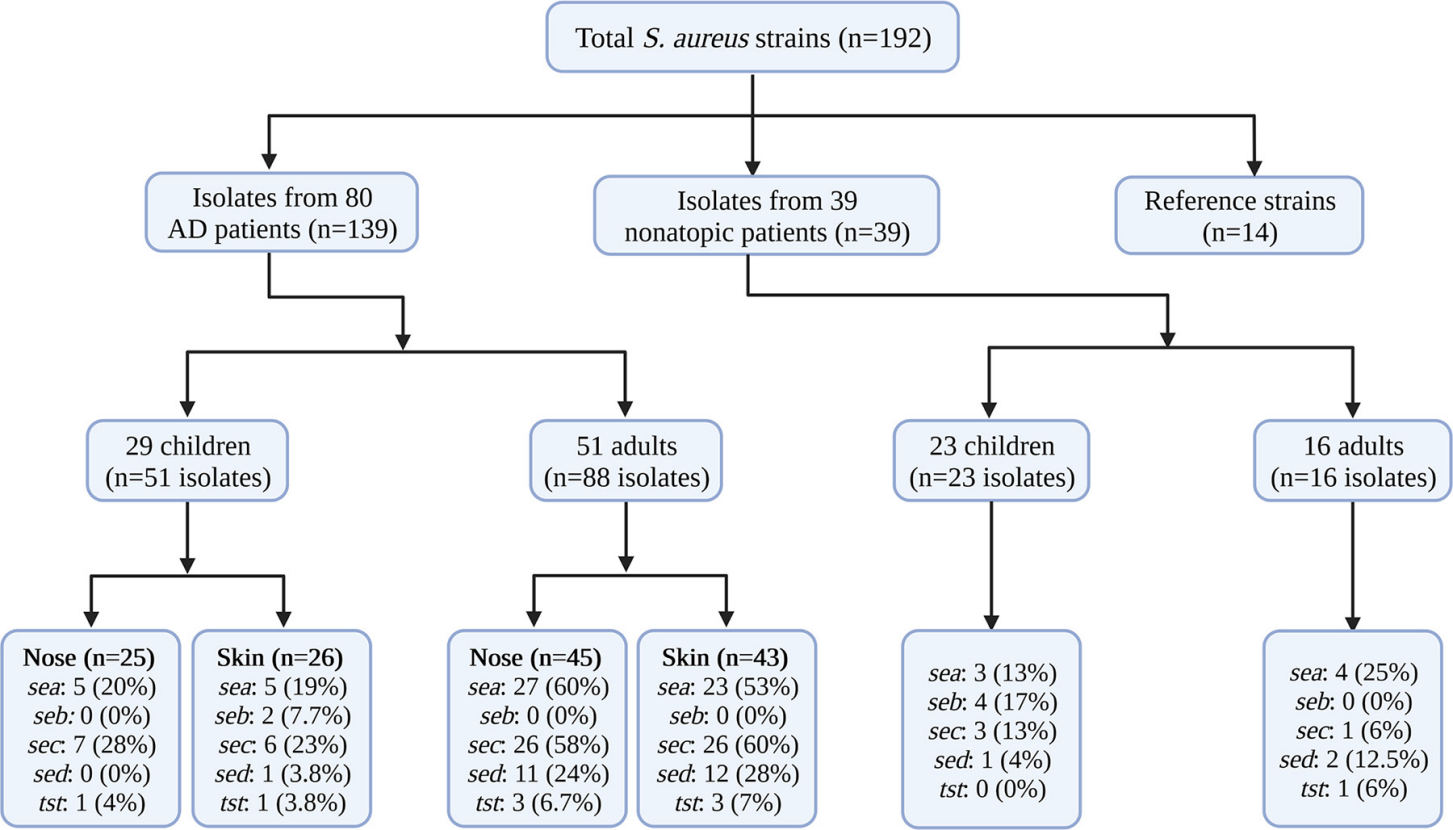
Diagram showing groups of S. aureus isolates tested by age group and number of toxin-encoding genes.

One hundred AD patients from the Department of Dermatology, Venereology and Allergology at the Medical University of Gdańsk, Poland, were sampled in 2014 and 2015 under the local Research Ethics Board (approval no. NKBBN/242-477/2014). Atopic dermatitis was diagnosed following the criteria of Hanifin and Rajka (18), which include pruritus, typical morphology and distribution of eczematous lesions, chronicity of the disease and personal or family history of atopy. From each patient, two samples were taken: a swab from the nose (N) and a swab from the skin (S). These 200 samples were cultured on solid medium to allow S. aureus growth. We obtained bacterial growth on trypticase soy broth (TSB) agar for 139 (69.5% of all) samples that derived from 80 AD patients. In the case of 58 patients (116 clinical isolates, 83% of all grown isolates), we observed growth from both sampled sites, the nose (N) and skin (S). In the case of the remaining isolates, they were cultured from either the nose or the skin samples. All cultured AD isolates were tested on both the phenotypic and the molecular level (e.g., susceptibility testing, *spa* typing, and virulence gene detection). Fifty-nine isolates (42%) were classified as community-acquired S. aureus (CA-SA). An isolate was considered a CA-SA when it was isolated from an individual who did not have any of the following risk factors: isolation of S. aureus more than 48 h after admission to hospital; a history of hospitalization, surgery, dialysis, or long-term-care facility stay within 1 year of S. aureus culture date; presence of an indwelling catheter or a percutaneous device at the time of culture; or previous isolation of S. aureus. A consort diagram is included for easier tracking of the strains and patients from which they were isolated (Fig. 1).

### Antimicrobial susceptibility among the population of AD isolates.

We chose several antibiotics that, among others, are used to treat soft tissue infections to check the resistance profile in the studied population. Overall, in the population of 139 isolates, resistance to erythromycin (39%), tetracycline (22%), ciprofloxacin (14.5%), mupirocin (10%), and fusidic acid (6.5%) was detected. In contrast, no resistance to daptomycin in the presence of Ca^2+^, linezolid, or vancomycin was detected. In addition, intermediate resistance was detected for mupirocin (6.5%), erythromycin (2.9%), and tetracycline (2.2%) (Table 1). Five isolates (3.5%) were identified as methicillin-resistant S. aureus (MRSA), and the presence of the *mec*A gene in these isolates was confirmed. Within the MRSA isolates, resistance to tetracycline (100%), erythromycin (100%), ciprofloxacin (80%), and fusidic acid (40%) was found.

**TABLE 1:** Characteristics, antibiotic resistance and presence of genes encoding staphylococcal superantigens and Panton-Valentine leukocidin genes in the Staphylococcus aureus strains used in the study^*a*^

Isolate name	Patient	CA/HA	MRSA/ MSSA	CIP	DAP (Ca^2^*)	E	FA	LZD	MUP	TET	Va		*Spa* type	Predicted MLST-CC/ST	Presence of SAg genes					
001N	A	HA	MRSA	R	S	R	R	S	S	R	S		t437	CC59						***pvl***
001S	A	HA	MRSA	R	S	R	R	S	S	R	S		t010	CC5						
002N	A	HA	MSSA	S	S	R	S	S	S	S	S		t005	CC22			***sec***	***sed***		
002S	A	HA	MSSA	S	S	R	S	S	S	S	S		t005	CC22			***sec***	***sed***		
003N	A	HA	MSSA	S	S	R	S	S	S	S	S		t091	CC7	***sea***		***sec***	***sed***		
003S	A	HA	MSSA	S	S	S	S	S	S	S	S		t091	CC7	***sea***		***sec***	***sed***		
004N	A	HA	MSSA	S	S	R	S	S	S	S	S		t084	CC15				***sed***		
004S	A	HA	MSSA	S	S	R	S	S	S	S	S		t084	CC15				***sed***		
005N	A	HA	MSSA	S	S	S	S	S	S	S	S		t2223	CC45			***sec***		***tsst-1***	
005S	A	HA	MSSA	S	S	S	S	S	S	S	S		t2223	CC45			***sec***		***tsst-1***	
006N	A	CA	MSSA	S	S	S	S	S	S	S	S		t1685	CC7	***sea***			***sed***		
006S	A	CA	MSSA	S	S	S	R	S	S	S	S		t091	CC7	***sea***			***sed***		
007N	A	HA	MSSA	S	S	S	S	S	S	S	S		t1255	CC398	***sea***			***sed***		
007S	A	HA	MSSA	S	S	S	S	S	S	S	S		t1255	CC398	***sea***			***sed***		
008N	A	CA	MSSA	S	S	S	S	S	S	S	S		t095	CC45	***sea***		***sec***	***sed***		
009S	A	CA	MSSA	R	S	S	S	S	S	S	S		t095	CC45			***sec***			
010N	A	HA	MSSA	S	S	S	S	S	S	S	S		t005	CC22						
010S	A	HA	MSSA	S	S	S	S	S	S	S	S		t091	CC7	***sea***					
011N	A	CA	MSSA	S	S	S	S	S	S	S	S		t1312	ST7425	***sea***		***sec***	***sed***		
011S	A	CA	MSSA	S	S	S	S	S	S	S	S		t1312	ST7425	***sea***		***sec***	***sed***		
012N	A	CA	MSSA	S	S	R	S	S	S	R	S		t024	CC8	***sea***			***sed***		
012S	A	CA	MSSA	S	S	R	S	S	S	R	S		t024	CC8	***sea***			***sed***		
013S	A	HA	MSSA	S	S	R	S	S	S	S	S		t024	CC8	***sea***			***sed***		
014S	A	CA	MSSA	S	S	S	S	S	S	S	S		t084	CC15						
015N	A	CA	MSSA	S	S	R	S	S	S	S	S		t018	CC30	***sea***			***sed***		
016N	A	HA	MSSA	S	S	S	S	S	S	S	S		t084	CC15			***sec***			
016S	A	HA	MSSA	S	S	R	S	S	S	S	S		t084	CC15			***sec***			
017N	A	CA	MSSA	S	S	R	S	S	S	S	S		t018	CC30	***sea sea***					
017S	A	CA	MSSA	S	S	R	S	S	S	S	S		t084	CC15	***sea***		***sec***	***sed***		
018N	A	CA	MSSA	S	S	R	S	S	S	S	S		t084	CC15	***sea***		***sec***			
018S	A	CA	MSSA	S	S	R	S	S	S	S	S		t880	CC45	***sea***		***sec***	***sed***		
019S	A	CA	MSSA	S	S	S	S	S	S	S	S		t050	CC45	***sea***		***sec***			
020N	A	CA	MSSA	S	S	S	S	S	S	S	S		t156	CC12	***sea***		***sec***	***sed***		
020S	A	CA	MSSA	S	S	S	S	S	S	S	S		t156	CC12	***sea***		***sec***	***sed***		
021S	C	HA	MSSA	S	S	R	S	S	S	R	S		t282	CC45	***sea sea***			***sed***		
022N	A	CA	MSSA	R	S	S	S	S	S	S	S		t091	CC7						
023N	C	HA	MSSA	S	S	S	R	S	S	R	S		t084	CC15						
023S	C	HA	MSSA	S	S	S	R	S	S	R	S		t084	CC15						
024S	A	CA	MSSA	S	S	S	S	S	S	S	S		t084	CC15						
025N	A	CA	MSSA	S	S	S	S	S	S	S	S		t091	CC7	***sea***		***sec***	***sed***		
025S	A	CA	MSSA	S	S	S	S	S	S	S	S		t091	CC7	***sea***		***sec***	***sed***		
026N	C	CA	MSSA	S	S	R	S	S	S	S	S		t015	CC45			***sec***			
027N	C	HA	MSSA	S	S	S	S	S	S	S	S		t091	CC7						
027S	C	HA	MSSA	S	S	S	S	S	S	S	S		t091	CC7						
028S	C	CA	MSSA	S	S	S	S	S	S	S	S		t282	CC45						
029N	A	HA	MSSA	S	S	R	S	S	S	R	S		t091	CC7	***sea***		***sec***			
029S	A	HA	MSSA	S	S	S	S	S	S	S	S		t091	CC7	***sea***		***sec***			
030N	C	HA	MSSA	R	S	S	S	S	S	S	S		t12375	CC97						
030S	C	HA	MSSA	S	S	S	S	S	S	S	S		t091	CC7						
031N	C	HA	MSSA	S	S	S	S	S	S	S	S		t12375	CC97						
031S	C	HA	MSSA	R	S	S	S	S	S	S	S		t015	CC45			***sec***			
032N	A	CA	MSSA	S	S	S	S	S	S	S	S		t12375	CC97	***sea***					
033N	A	HA	MSSA	S	S	R	S	S	S	S	S		t12375	CC97	***sea***		***sec***			
033S	A	HA	MSSA	R	S	R	S	S	S	S	S		t12375	CC97	***sea***		***sec***			
034N	C	HA	MSSA	S	S	I	R	S	S	S	S		t12375	CC97						
034S	C	HA	MSSA	S	S	S	S	S	S	S	S		t091	CC7						
035N	A	HA	MSSA	S	S	S	S	S	S	S	S		t050	CC45			***sec***			
035S	A	HA	MSSA	S	S	S	S	S	S	S	S		t050	CC45			***sec***			
036N	A	HA	MSSA	S	S	R	S	S	S	S	S		t015	CC45	***sea***		***sec***			
036S	A	HA	MSSA	S	S	R	S	S	R	S	S		t015	CC45	***sea***		***sec***			
037N	C	CA	MSSA	S	S	S	S	S	S	S	S		t864	CC10	***sea***		***sec***			
037S	C	CA	MSSA	S	S	S	S	S	S	S	S		t12375	CC97						
038N	C	HA	MSSA	S	S	S	S	S	R	S	S		t091	CC7						
038S	C	HA	MSSA	S	S	S	S	S	R	S	S		t091	CC7						
039N	A	CA	MSSA	S	S	R	S	S	S	S	S		t2301	CC45			***sec***			
039S	A	CA	MSSA	S	S	S	S	S	R	S	S		t091	CC7	***sea***		***sec***			
040N	A	CA	MSSA	S	S	S	S	S	S	R	S		t091	CC7	***sea***		***sec***			
040S	A	CA	MSSA	S	S	S	S	S	S	R	S		t2301	CC45						
042N	A	CA	MSSA	S	S	S	S	S	R	S	S		t127	CC1	***sea***		***sec***			
043S	C	CA	MRSA	S	S	R	S	S	S	R	S		t437	CC59	***sea***	***seb seb***	***sec***			***pvl***
044N	A	CA	MSSA	S	S	R	S	S	S	R	S		t127	CC1	***sea***		***sec***			
045N	C	HA	MSSA	I	S	S	S	S	R	R	S		t015	CC45			***sec***			***pvl***
045S	C	HA	MSSA	I	S	S	S	S	I	R	S		t015	CC45			***sec***			***pvl***
046N	A	HA	MSSA	S	S	R	S	S	S	R	S		t084	CC15	***sea***		***sec***			
046S	A	HA	MSSA	S	S	R	S	S	S	S	S		t359	CC97	***sea***		***sec***			
048N	C	HA	MSSA	S	S	R	S	S	S	S	S		t5995	CC22						***pvl***
048S	C	HA	MSSA	S	S	S	S	S	S	S	S		t5995	CC22						***pvl***
051N	A	CA	MSSA	S	S	S	S	S	S	S	S		t692	CC88						***pvl***
051S	A	CA	MSSA	S	S	R	S	S	R	S	S		t091	CC7			***sec***			
052N	C	CA	MSSA	S	S	S	S	S	S	S	S		t091	CC7						
052S	C	CA	MSSA	R	S	R	S	S	S	I	S		t091	CC7						***pvl***
053N	A	HA	MSSA	S	S	S	S	S	S	S	S		t091	CC7	***sea***		***sec***			
053S	A	HA	MSSA	S	S	R	S	S	S	S	S		t091	CC7	***sea***		***sec***			
055N	A	HA	MSSA	R	S	S	S	S	S	S	S		t537	CC72			***sec***			
055S	A	HA	MSSA	R	S	S	S	S	S	S	S		t537	CC72			***sec***			
056N	C	HA	MSSA	S	S	R	S	S	R	S	S		t209	CC9			***sec***			***pvl***
056S	C	HA	MSSA	I	S	R	S	S	R	S	S		t005	CC22						***pvl***
057N	A	CA	MSSA	R	S	S	S	S	S	S	S		t267	CC97	***sea***					
057S	A	CA	MSSA	S	S	S	S	S	S	R	S		t267	CC97	***sea***					
059N	A	CA	MSSA	S	S	S	S	S	S	S	S		t091	CC7						
059S	A	CA	MSSA	R	S	S	S	S	S	S	S		t537	CC72			***sec***			
060N	C	HA	MSSA	S	S	R	S	S	S	S	S		t880	CC45						
060S	C	HA	MSSA	S	S	S	S	S	S	S	S		t091	CC7						
061N	A	HA	MSSA	S	S	S	S	S	S	S	S		t1814	CC88	***sea***		***sec***			
061S	A	HA	MSSA	S	S	I	S	S	S	S	S		t692	CC88						
062N	C	HA	MRSA	R	S	R	S	S	S	R	S		t008	CC8	***sea***					
062S	C	HA	MRSA	R	S	R	S	S	S	R	S		t008	CC8	***sea***					
063N	A	HA	MSSA	S	S	S	S	S	S	S	S		t1743	CC7	***sea***		***sec***			
063S	A	HA	MSSA	S	S	R	S	S	S	S	S		t1743	CC7	***sea***		***sec***			
064S	A	HA	MSSA	S	S	S	S	S	S	S	S		t012	CC30	***sea***		***sec***			
065N2	C	HA	MSSA	S	S	S	S	S	S	S	S		t537	CC72						
065N3	C	HA	MSSA	S	S	S	S	S	S	S	S		t091	CC7						
065S	C	HA	MSSA	S	S	S	R	S	S	S	S		t156	CC12			***sec***			***pvl***
068N	A	HA	MSSA	S	S	R	S	S	S	S	S		t012	CC30					***tsst-1***	
068S	A	HA	MSSA	I	S	S	S	S	S	R	S		t012	CC30					***tsst-1***	
070N	C	HA	MSSA	S	S	R	S	S	S	R	S		t015	CC45			***sec***			***pvl***
070S	C	HA	MSSA	S	S	R	S	S	R	R	S		t015	CC45			***sec***			
071N	C	HA	MSSA	R	S	R	S	S	S	S	S		t015	CC45	***sea***		***sec***			
071S	C	HA	MSSA	I	S	S	S	S	R	S	S		t309	CC22						
072N	A	HA	MSSA	S	S	S	S	S	S	S	S		t091	CC7						
072S	A	HA	MSSA	S	S	S	S	S	S	S	S		t091	CC7						
073N	C	HA	MSSA	S	S	R	S	S	S	S	S		t693	CC1						
073S	C	HA	MSSA	S	S	S	S	S	S	S	S		t091	CC7						
074N	A	HA	MSSA	S	S	S	S	S	S	S	S		t156	CC12			***sec***			
074S	A	HA	MSSA	S	S	S	S	S	S	I	S		t156	CC12			***sec***			
077N	A	HA	MSSA	S	S	S	S	S	S	S	S		t537	CC72			***sec***			
077S	A	HA	MSSA	S	S	S	S	S	S	S	S		t537	CC72			***sec***			
079N	A	CA	MSSA	S	S	R	S	S	S	S	S		t20244	ST1027						***pvl***
080N	A	CA	MSSA	S	S	R	S	S	S	S	S		t230	CC45	***sea***		***sec***			
080S	A	CA	MSSA	S	S	R	S	S	S	S	S		t1442	CC718						
081N	A	CA	MSSA	S	S	I	S	S	S	S	S		t18582	CC30	***sea***				***tsst-1***	
081S	A	CA	MSSA	I	S	R	S	S	R	R	S		t18582	CC30	***sea***				***tsst-1***	
083N	A	CA	MSSA	S	S	S	S	S	S	S	S		t084	CC15						
083S	A	CA	MSSA	S	S	S	S	S	S	S	S		t084	CC15						
086N	C	CA	MSSA	S	S	R	S	S	I	S	S		t693	CC1						***pvl***
086S	C	CA	MSSA	R	S	R	S	S	I	S	S		t091	CC7						
087N	C	CA	MSSA	S	S	R	S	S	S	S	S		t359	CC97						***pvl***
088N	C	HA	MSSA	S	S	R	S	S	I	R	S		t056	CC101	***sea***		***sec***			
089N	C	CA	MSSA	S	S	S	S	S	R	S	S		t091	CC7						
089S	C	CA	MSSA	S	S	S	S	S	R	R	S		t091	CC7						***pvl***
090S	C	CA	MSSA	S	S	I	R	S	S	R	S		t160	CC12	***sea***	***seb***	***sec***			
092N	A	HA	MSSA	S	S	S	S	S	I	S	S		t091	CC7	***sea***					***pvl***
093N	A	HA	MSSA	R	S	S	S	S	S	I	S		t521	CC97	***sea***		***sec***			
093S	A	HA	MSSA	I	S	S	S	S	S	R	S		t521	CC97	***sea***		***sec***			
094N	C	CA	MSSA	S	S	S	S	S	S	S	S		t2301	CC45						***pvl***
094S	C	CA	MSSA	S	S	R	S	S	I	S	S		t2301	CC45						
095S	C	CA	MSSA	S	S	R	S	S	I	R	S		t037	CC30	***sea***				***tsst-1***	
096N	C	CA	MSSA	R	S	S	S	S	I	R	S		t021	CC30	***sea***				***tsst-1***	
096S	C	CA	MSSA	R	S	S	S	S	I	R	S		t867	CC7						

a N, isolate from a nose; S, isolate from skin; A, adult; C, child; CA, community acquired; HA, hospital acquired; MRSA, methicillin-resistant Staphylococcus aureus; MSSA, methicillin-sensitive Staphylococcus aureus; CIP, ciprofloxacin; DAP, daptomycin; E, erythromycin; FA, fusidic acid; LZD, linezolid; MUP, mupirocin; TET, tetracyclin; Va, vancomycin; CC/ST, clonal complex/sequence type based on MLST; the designation “t” with a number (e.g., t091) indicates a specific type of *spa* gene according to Ridom nomenclature (http://spaserver.ridom.de/); SAg, superantigens. *sea*, *seb*, *sec*, *sed*, and *tsst-1* = staphylococcal enterotoxin A, B, C, D, and toxic shock syndrome toxin 1, respectively; *pvl* = *lukS*-*PV* and *lukF*-*PV* genes encoding Panton-Valentine leukocidin.

Apart from the identified MRSA isolates, no multidrug resistance was detected among the methicillin-sensitive S. aureus (MSSA) isolates.

### Staphylococcal enterotoxin and *tsst-1* gene distribution among the S. aureus AD patient isolates.

We studied the presence of genes encoding staphylococcal enterotoxins (SEs) A, B, C, D, and toxic shock syndrome toxin-1 (TSST-1) (Table 1). According to the data obtained, 65% of all studied isolates contained at least one of the studied genes. In the MRSA group, this percentage accounted for 60%, which was lower than the analogous percentage in the MSSA group (66%). The most prevalent gene in the whole studied population was the *sec* gene (confirmed in 65 isolates), followed by *sea* (60), *sed* (24), and *tsst*-*1* (8). Only 2 *seb* genes were detected in all the isolates collected.

The analysis of the SAg gene distribution among adults and children revealed interesting differences (Table 2 and 3). The detected frequencies of *sea*, *sec*, *sed*, and *tsst*-*1* in both groups differed significantly and were higher in the adult group. The frequency of detection of these genes was approximately twice that of children. In the case of the *sed* gene, the vast majority, namely, 23 out of 24 genes, were detected in the adult group, thus indicating that it was characteristic of the adult population. We found only two *seb* genes exclusively in the pediatric group. These differences were not affected by the MRSA distribution, as it was underrepresented in both groups: 3 isolates in children and 2 isolates in adults. We observed another interesting difference between children and adult isolates. In the group of children, most strains only had one out of the studied genes (10 strains, 1 gene; 6 strains, 2 genes; 2 strains, 3 genes), while in the adult group, most strains had > 1 studied gene (48 strains, 2 or 3 genes; 24 strains, 1 gene).

**TABLE 2:** Frequency of SAg gene detection in S. aureus isolated from children with atopic dermatitis^*a*^

Study group	Staphylococcal genes				
	*sea*	*seb*	*sec*	*sed*	*tsst-1*
Children (*n* = 51)	10 (19.6%)	2 (4%)	13 (25.5%)	1 (2%)	2 (4%)
CA (*n* = 18)	5 (27.7%)	2 (11%)	4 (22%)	0 (0%)	2 (11%)
HA (*n* = 33)	5 (15%)	0 (0%)	9 (27%)	1 (3%)	0 (0%)
Nose (*n* = 25)	5 (20%)	0 (0%)	7 (28%)	0 (0%)	1 (4%)
Skin (*n* = 26)	5 (19%)	2 (7.7%)	6 (23%)	1 (3.8%)	1 (3.8%)
MRSA (*n* = 3)	3 (100%)	1 (33.3%)	1 (33.3%)	0 (0%)	0 (0%)
MSSA (*n* = 48)	7 (14.5%)	1 (2%)	12 (25%)	1 (2%)	2 (4%)

a CA, community acquired; HA, hospital acquired; MRSA, methicillin-resistant Staphylococcus aureus; MSSA, methicillin-sensitive Staphylococcus aureus.

**TABLE 3:** Frequency of SAg gene detection in S. aureus isolated from adults with atopic dermatitis^*a*^

Study group	Staphylococcal genes				
	*sea*	*seb*	*sec*	*sed*	*tsst-1*
Adults (*n* = 88)	50 (57%)	0 (0%)	52 (59%)	23 (26%)	6 (7%)
CA (*n* = 41)	27 (66%)	0 (0%)	20 (49%)	14 (34%)	2 (5%)
HA (*n* = 47)	23 (49%)	0 (0%)	32 (68%)	9 (19%)	4 (8.5%)
Nose (*n* = 45)	27 (60%)	0 (0%)	26 (58%)	11 (24%)	3 (6.7%)
Skin (*n* = 43)	23 (53%)	0 (0%)	26 (60%)	12 (28%)	3 (7%)
MRSA (*n* = 2)	0 (0%)	0 (0%)	0 (0%)	0 (0%)	0 (0%)
MSSA (*n* = 86)	50 (58%)	0 (0%)	52 (60%)	23 (27%)	6 (7%)

a CA, community acquired; HA, hospital acquired; MRSA, methicillin-resistant Staphylococcus aureus; MSSA, methicillin-sensitive Staphylococcus aureus.

In a group of S. aureus isolated from nonatopic controls, the distribution of SAg genes exhibited a different profile: *sea*, *sec*, *sed*, and *tsst*-1 were detected less frequently than in the AD group (Table 4, Table S1). In contrast to the AD group, the *sea*, *sec*, and *tsst*-*1* genes were less frequent and the *seb* gene was more frequent in nonatopic controls. It must be emphasized, however, that nonatopic isolates were collected in 2008 and 2009, whereas the current AD isolates were collected in 2014 and 2015.

**TABLE 4:** Frequency of SAg gene detection in S. aureus isolated from nonatopic patients

Nonatopic patients	Staphylococcus aureus genes				
	*sea*	*seb*	*sec*	*sed*	*tsst-1*
MSSA (*n* = 39)	7 (17.9%)	4 (10.3%)	4 (10.3%)	3 (7.7%)	1 (2.6%)

An interesting observation was the detection of numerous isolates carrying *lukS*/*lukF*-*PV* genes (*pvl*-genes) encoding the Panton-Valentine leukocidin (PVL) (18/139; 12.9%). Most of the detected genes came from isolates from children (14/18; 77.7%). In the group of 18 *pvl*-positive isolates, 11 isolates were from the nose and 7 (from children only) from the skin. In 11 out of 18 *pvl*-positive isolates, the *lukS/lukF*-*PV* genes were the only detected ones among the genes tested. In two MRSA isolates (CC59-t437) originating from two different patients, the presence of genes coding for PVL was also detected (Table 1).

The distribution of SAg genes among nasal isolates versus skin isolates revealed no statistically significant differences. The *sea*, *sec*, *sed*, and *tsst*-*1* genes were detected with a similar frequency in nasal isolates and skin isolates, which was apparent for both groups studied, children and adults. For a more specific comparison, we excluded isolates from the same patient that belonged to the same *spa* type; however, in this case, no significant differences were observed.

### Staphylococcal toxin production.

To test whether the strains that possess genes encoding SAgs can produce the proteins, we performed immunodetection of SAgs during the logarithmic phase of growth in randomly chosen isolates. On average, staphylococcal enterotoxin A (SEA) and staphylococcal enterotoxin C (SEC) were produced in the largest amounts. From the experiments performed, we observed no difference between skin and nose isolates (Table 5). We did not observe any difference in the amount of toxins produced depending on the origin of the isolates (hospital acquired versus community acquired) or the age of the patients from whom the isolates were derived (children versus adults). We observed that 3 MRSA isolates had *sea* genes. Two of the 3 were from the same patient and belonged to the same *spa* type (t008). Interestingly, the third of the above-mentioned MRSA isolates, apart from SEA, also produced SEB, which was detected in only two cases in the whole population, and the strain also produced SEC.

**TABLE 5:** Comparative characterization of staphylococcal superantigen production by randomly selected nasal and skin isolates carrying the *sea*, *seb*, *sec*, *sed*, or *tsst-1* gene

Protein	Nasal isolates	Skin isolates	Nonatopic isolates	Total	*P* value
SEA	*n* = 8	*n* = 11	*n* = 6	*n* = 25	0.1526^*a*^
Avg. (SD)	8.4 (7.4)	5.7 (8.3)	1.2 (0.9)	5.5 (7.3)	
Range	0.3–20.2	0.1–25.0	0.2–2.4	0.1–25.0	
Median	7.8	0.7	1.0	2.0	
95% CI	[2.2; 14.6]	[0.1; 11.3]	[0.2; 2.1]	[2.5; 8.5]	
SEB	*n* = 0	*n* = 1	*n* = 4	*n* = 5	-
Avg. (SD)		8.1 (0.0)	3.9 (3.5)	4.8 (3.5)	
Range		8.1–8.1	0.1–7.5	0.1–8.1	
Median		8.1	4.0	6.1	
95% CI		[0.0; 0.0]	[−1.6; 9.4]	[0.4; 9.1]	
SEC	*n* = 12	*n* = 16	*n* = 4	*n* = 32	0.2165^*a*^
Avg. (SD)	13.9 (22.9)	6.0 (13.6)	0.7 (0.3)	8.3 (17.2)	
Range	0.2–61.1	0.0–55.1	0.3–1.1	0.0–61.0	
Median	2.9	1.5	0.7	1.5	
95% CI	[−0.6; 28.4]	[−1.2; 13.3]	[0.1; 1.2]	[2.1; 14.5]	
SED	*n* = 3	*n* = 7	*n* = 3	*n* = 13	0.125^*a*^
Avg. (SD)	2.3 (0.8)	1.2 (0.5)	1.1 (0.2)	1.4 (0.7)	
Range	1.7–3.2	0.6–2.0	0.9–1.3	0.6–3.2	
Median	2.0	1.1	1.0	1.2	
95% CI	[0.3; 4.3]	[0.7; 1.7]	[0.6; 1.5]	[1.0; 1.8]	
TSST-1	*n* = 4	*n* = 2	*n* = 1	*n* = 7	0.8170^*b*^
Avg. (SD)	3.0 (1.7)	3.4 (2.7)	0.3 (0.0)	2.7 (1.9)	
Range	1.6–4.7	1.5–5.2	0.3 to 0.3	0.3–5.2	
Median	2.9	3.4	0.3	1.6	
95% CI	[0.3; 5.7]	[−20.5; 27.2]	[0.0; 0.0]	[0.9; 4.5]	

a Kruskal-Wallis test; the values of protein concentration are in μg/mL.

b Mann-Whitney U test; the values of protein concentration are in μg/mL.

### Identification of the clonal structure of S. aureus isolates.

**FIG 2:**
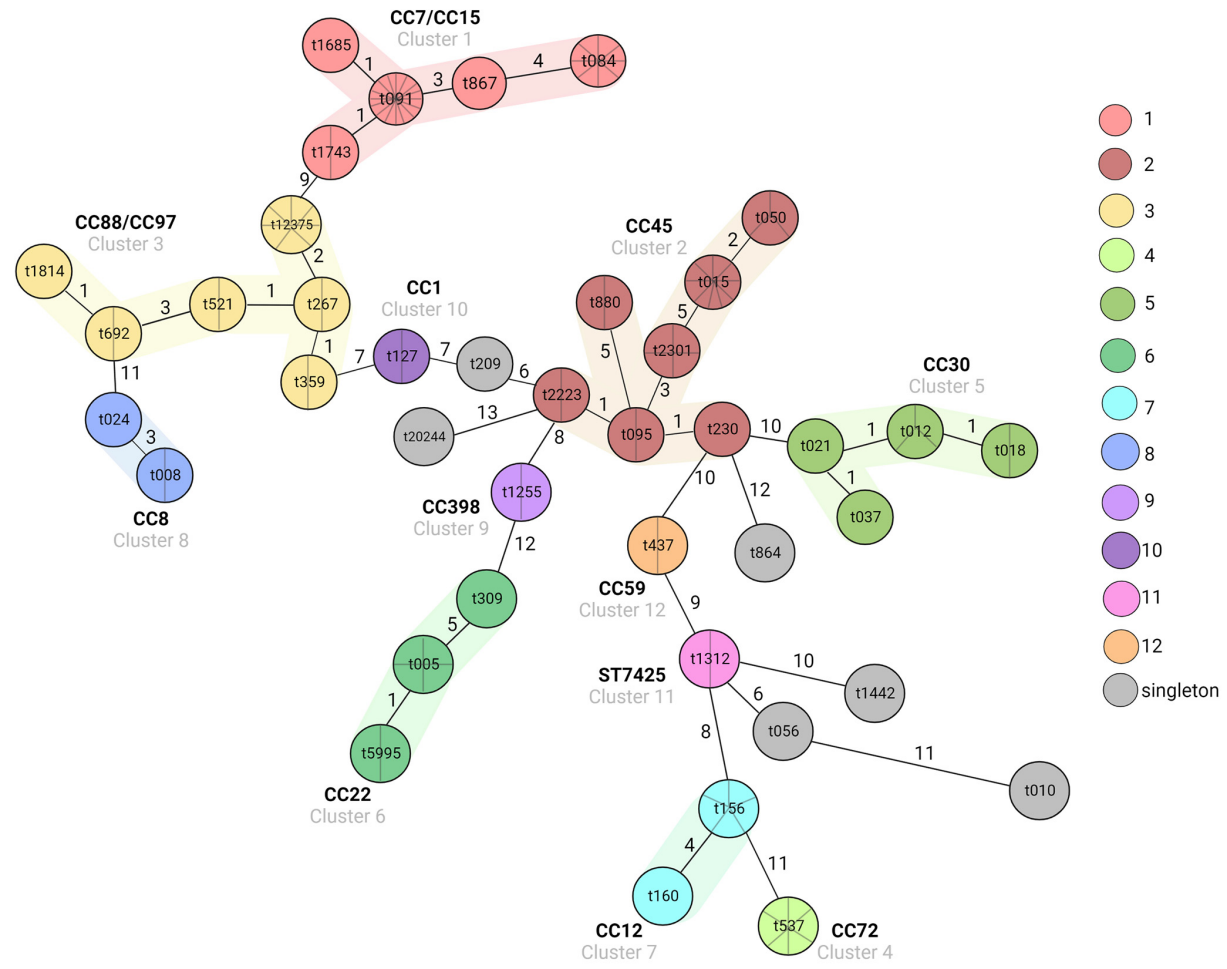
A minimum spanning tree (MST), created using Ridom SeqSphere+ software, based on *spa* type profiles of 133 S. aureus isolates recovered from AD patients with MLST-CCs/STs indicated.

We obtained 43 different *spa* types among the studied population, including one new type (t20244). Using Ridom SeqSphere+ software, we assigned the *spa* types to 12 clusters, including four clusters consisting of two isolates of the same *spa* type, each, and six singletons. Three *spa* types (t282, t639, and 18582), shorter than 5 repeats, were excluded from the analysis (Fig. 2).

The most often represented type was t091, which comprised 32 isolates (23% of all isolates), followed by t084 (9.5%), t015 (6.5%), t12375 (5%), t537 (4.3%), t156 (3.6%), t2301 (2.9%), and t005 (2.9%). The remaining *spa* types were less frequent and were represented by 3, 2, or 1 isolates. The identified *spa* types could be assigned to 18 MLST-clonal complexes (CCs): CC7 (36 isolates), CC45 (25 isolates), CC97 and CC15 (13 isolates each), CC30 (9 isolates), CC22 (7 isolates), CC12 and CC72 (6 isolates each), and CC8 (5 isolates). The remaining CCs were represented by 1 to 4 isolates. The first four CCs: CC7, CC45, CC97, and CC15 constituted 62.5% of all isolates. Isolates of *spa* types t1312 and t20244 belonged to ST7425 and ST1027, respectively, which were not assigned to any CC.

The identified MRSA isolates (*n* = 5) were grouped into 3 various CCs, CC59 (2 isolates), CC8 (2 isolates), and CC5 (1 isolate), with 3 *spa* types, t437, t008, and t010, respectively.

Brackets indicate the percentage of isolates with a gene detected within a given *spa* type (%); numbers indicating percentage of isolates with a gene detected within a given clonal complex are in boldface; n denotes the number of genes detected in the whole population studied (139 isolates); the designation CC denotes a clonal complex; and the designation t with a number (e.g., t091) indicates a specific type of *spa*, according to Ridom nomenclature (http://spaserver.ridom.de/).

Analysis of the relationship between the frequency of genes and the type of MLST-CC and the type of *spa* revealed that the distribution of the frequency of occurrence of a given gene differs in a statistically significant way depending on the *spa*/MLST-CC type (Table 6). For all genes (*sea*, *seb*, *sec*, *sed* and *tsst-1*) there is a statistically significant correlation with the type of *spa*/MLST-CC (statistical significance in this case shows that the frequency distribution of a given gene differs significantly with respect to the *spa* type and MLST-CC type).

**TABLE 6:** Analysis of the relationship between the frequency of the genes *sea*, *seb*, *sec*, *sed* and *tsst*-*1* and the MLST-CC/ST and *spa* type

MLST-CC/ST	*Spa* type	*Sea* (*n* = 60)	*Seb* (*n* = 2)	*Sec* (*n* = 65)	*Sed* (*n* = 24)	*Tsst-1* (*n* = 8)
CC7		16 (26.67)		13 (20)	6 (24,93)	
	t091	13 (21.67)		11 (16.92)	5 (20.83)	
	t1743	2 (3.33)		2 (3.08)		
	t1685	1 (1.67)			1 (4.17)	
CC45		8 (13.35)		16 (24.63)	3 (12.51)	2 (25)
	t015	3 (5)		9 (13.85)		
	t2301			1 (1.54)		
	t050	1 (1.67)				
	t095	1 (1.67)		2 (3.08)	1 (4.17)	
	t282	1 (1.67)			1 (4.17)	
	t880	1 (1.67)		1 (1.54)	1 (4.17)	
	t2223			2 (3.08)		2 (25)
	t230	1 (1.67)		1 (1.54)		
CC15						
	t084	3 (5)		5 (7.69)	3 (12.5)	
CC97		8 (13.33)		5 (7.7)		
	t12375	3 (5)		2 (3.08)		
	t267	2 (3.33)				
	t359	1 (1.67)		1 (1.54)		
	t521	2 (3.33)		2 (3.08)		
CC30		7 (11.67)		1 (1.54)	1 (4.17)	6 (75)
	t012	1 (1.67)		1 (1.54)		2 (25)
	t018	2 (3.33)			1 (4.17)	
	t021	1 (1.67)				1 (12.5)
	t037	1 (1.67)				1 (12.5)
	t18582	2 (3.33)				2 (25)
CC22						
	t005			2 (3.08)	2 (8.33)	
CC12		3 (8.33)	1 (50)	6 (9.23)	2 (8.33)	
	t156	2 (3.33)		5 (7.69)	2 (8.33)	
	t160	1 (1.67)	1 (50)	1 (1.54)		
CC8		5 (8.33)			3 (12.5)	
	t024	3 (5)			3 (12.5)	
	t008	2 (3.33)				
CC72						
	t537			5 (7.69)		
CC1						
	t127	2 (3.33)		2 (3.08)		
CC88						
	t1814	1 (1.67)		1 (1.54)		
CC59						
	t437	1 (1.67)	1 (50)	1 (1.54)		
CC398						
	t1255	2 (3.33)			2 (8.33)	
CC9						
	t209			1 (1.54)		
CC10						
	t864	1 (1.67)		1 (1.54)		
CC101						
	t056	1 (1.67)		1 (1.54)		
ST7425						
	t1312	2 (3.33)		2 (3.08)	2 (8.33)	

The *sea* gene was most often associated with *spa* type t091 in nearly 22% of *sea*-positive isolates, *spa* types t084, t024, t12375, and t015 in 5% each, and the rest in 2 to 3%. The *sec* gene was most often associated with *spa* type t091 in almost 17% of *sec*-positive isolates; *spa* type t015 in 14%; t156, t084, and t537 in 8% each; and the rest in 2 or 3% each. The *sed* gene was most often associated with *spa* type t091 in nearly 21% of *sed*-positive isolates; *spa* types t084 and t024 in 13% each; t005, t1255, t1312, and t156 in 8% each; and the rest in 4% each. In over half (62%) of the tested isolates, the presence of >1 gene was detected in a given isolate. The most common combinations were *sea* + *sec* (26 isolates), *sea* + *sed* (9 isolates), *sea* + *sec* + *sed* (11 isolates), and *sea* + *seb* + *sec* (2 isolates). The *tsst-1* gene was most often associated with the *spa* types t2223, t012, and t18582 in 25% of *tsst-1*-positive isolates, and the remaining ones in almost 13% each.

### The efficiency of aPDI against the studied clinical isolates.

**FIG 3:**
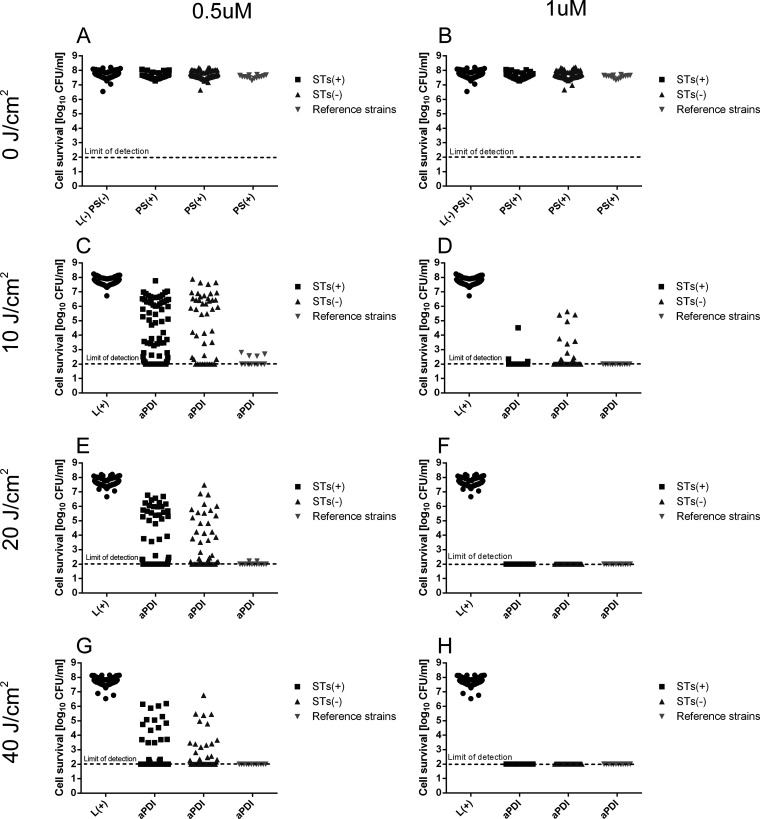
Photoinactivation of S. aureus atopic dermatitis isolates. Two concentrations of rose bengal RB: 0.5 μM (A, C, E, and G) and 1 μM (B, D, F, and H) were applied at increasing light doses in the range of 0 to 40 J/cm^2^ (as indicated on the left). The initial number of treated bacteria was 10^8^ CFU/mL for each isolate. A total of 139 clinical isolates were tested and 14 reference strains (see Materials and Methods for a detailed description). L(−)PS(−), untreated bacterial cell kept in the dark (0 J/cm^2^); PS(+), bacterial cells treated with RB alone and stored in the dark (0 J/cm^2^); L(+), bacterial cells treated with green light alone; aPDI, bacterial cells treated with RB and green light; STs, strains with the presence (+) or absence (−) of staphylococcal toxin genes.

To check the studied S. aureus population susceptibility to photoinactivation as a possible treatment option for atopic skin decolonization, we subjected the studied isolates to rose bengal (RB)-mediated phototreatment. We were interested in whether the genetic diversity observed in the studied population affects phototreatment efficacy. Specifically, we were interested in checking whether the strains carrying SAg-producing genes are equally sensitive to the applied phototreatment *in vitro*. Phototreatment of S. aureus isolates from adults and children indicated a very efficient reduction (≥3 log_10_ CFU/mL; 99.9%) in the viable count of all isolates. The great majority of the studied population (88.4%) was photoinactivated under the following mild conditions: 0.5 μM RB and 40 J/cm^2^. Increasing the concentration of RB to 1 μM resulted in 100% of strains being photoinactivated, even at half of the light dose (20 J/cm^2^) (Fig. 3). The obtained results indicate a very effective method of photoinactivation, which can be successfully used in research on more complex models.

**FIG 4:**
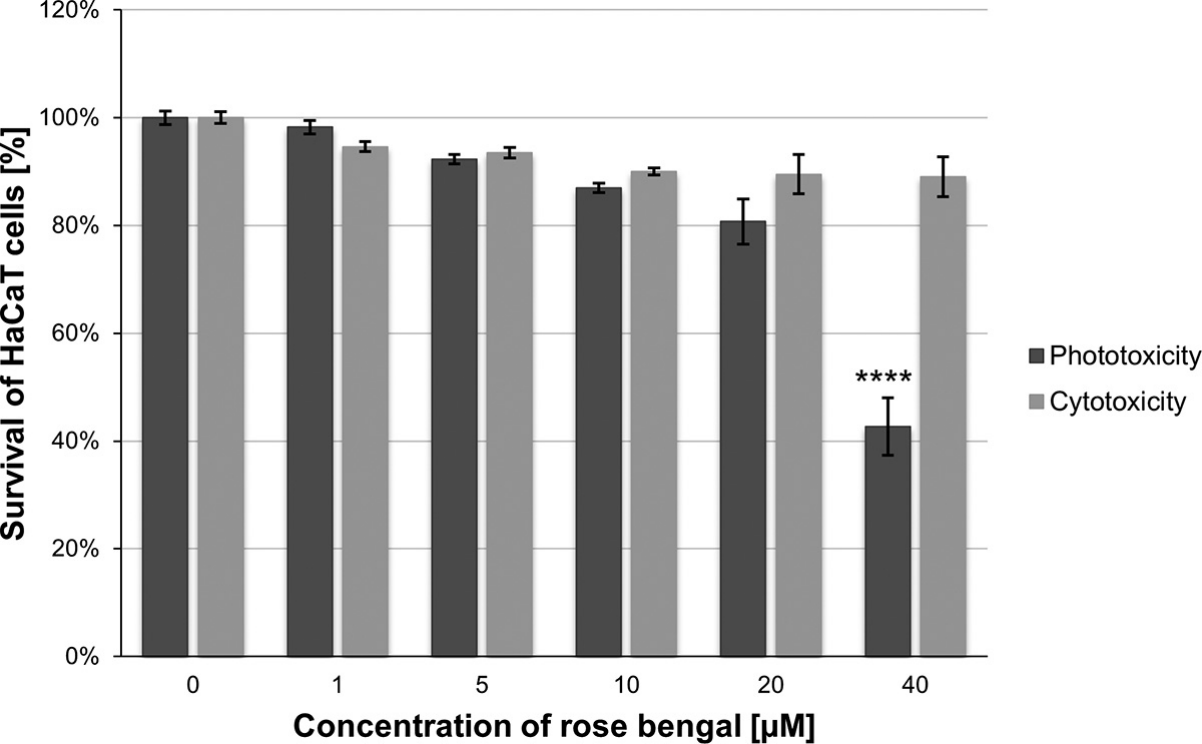
Photo- and cytotoxicity against the immortalized human cell line HaCaT. Photo- and cytotoxicity were measured using the MTT assay according to a standard protocol described in Materials and Methods. Relative toxicities are shown according to control cells cultured without the presence of a photosensitizer (rose bengal) and without light treatment. The statistical significance was measured using one-way analysis of variance (ANOVA) and Dunnett’s multiple-comparison test. Significance is marked with asterisks (****, *P* < 0.0001) with respect to untreated samples (cells kept in the dark).

The conditions of phototreatment that we applied to S. aureus isolates were also studied with respect to a keratinocyte cell line (HaCaT) to check the cyto- and phototoxicity in a eukaryotic model. Under the tested conditions of RB and light dose combinations, the photo- and cytotoxicity against the eukaryotic cell line were negligible. We also checked higher concentrations of RB under dark and light conditions, which resulted in only a slight decrease in keratinocyte survival (Fig. 4). This result means that aPDI is neither cyto- nor phototoxic against this cell line under the studied *in vitro* conditions.

## DISCUSSION

The epidemiology of staphylococcal infections is extremely diverse, with new clones emerging and spreading in different populations at geographically different locations. The isolation rate of S. aureus in the population of AD patients might be as high as 90% in some populations. Given the frequency of isolation of S. aureus from atopic skin as well as the high prevalence of AD (15 to 30% in children and 2 to 10% in adults worldwide) (19), such patients represent a rich reservoir of staphylococcal isolates and potential drug-resistant isolates (20).

Consequently, there is a continuing need to monitor the genetic variability of these isolates as well as their toxigenic properties. Literature data indicate that skin isolates of AD patients produce large amounts of extracellular toxins, including SAgs, which are the subject of this analysis. However, the results of our analyses showed tremendous heterogeneity in the level of protein production among various isolates; moreover, we observed no differences in the number of proteins produced by nose and skin isolates or isolates from nonatopic patients (Table 5). This could be because we used TSB medium for bacterial cultures, whereas the highest protein production was observed in bovine heart media (21, 22). In addition, a more thorough selection of the S. aureus study population based on the clinical presentation of AD (mild versus severe) in the patients from whom the isolates were obtained would be more informative with respect to the amount of toxin produced and the search for correlations. Staphylococcal enterotoxins (SE) are responsible for foodborne infections but also act as so-called superantigens that induce dysregulation of the immune response in AD patients. Data in the literature indicate that the type of inflammation and immune responses in AD patients depend on specific S. aureus strains (5). This shows that the diversity of AD strains is a factor that significantly contributes to the exacerbation of disease symptoms and encourages more in-depth research to identify the characteristics of S. aureus (including genetic attributes) that play a role in adaptation to the disease or to living on atopic skin. The fact that S. aureus frequently colonizes nasal epithelial cells without causing disease symptoms causes difficulties in epidemiological studies. On the other hand, S. aureus colonization of the skin is rather rare in the general population but very common in the AD population. The answer to the question of whether the S. aureus residing on the skin is genetically identical to that residing in the nasal epithelium of AD patients is not clearly defined. In previous studies, it was found that strains isolated from AD patients differ from those isolated from other sources: they induce a stronger immune response than nonatopic strains or common commensal Staphylococcus epidermidis (5, 23, 24), they produce important exogenous proteases (25), and they show distinct cell wall properties (26). By analyzing S. aureus at the genetic level by tracing the relationship based on an analysis of *spa* types, many authors have demonstrated a distribution of genotypes in an S. aureus population isolated from AD patients (17) that is heterogeneous but insufficiently sensitive to explain the underlying mechanism of the dependence between atopic skin and S. aureus presence. Undoubtedly, the SEs produced by S. aureus play an important role in this situation, but their distribution within clones is inconclusive, although SEs are related to certain clones (27).

A comparison of our results regarding the genetic structure in the studied population with data published in other countries on AD populations shows some differences. For example, CC1 has been shown in some studies to be the most frequently isolated genotype from AD patients (28, 29). In contrast, in our case, CC1 was detected sporadically, and CC7 was the most frequently isolated clone. We observed that the S. aureus population colonizing AD patients, studied at a genetic level based on commonly used molecular methods such as *spa* typing and MLST-CC, is consistent with the population observed in a given geographical area and is not specific to the type of disease. The most frequently detected genotype in our studied population was CC7 (ST7-t091), like the overall European trend (27, 30). It is worth noting, however, that the clone CC7 (ST7-t091) was present in our population with a 4-fold higher frequency (22.5% versus 5.3% or 5.8%) compared to that for bloodstream-isolated S. aureus or isolates circulating in the healthy carrier population (27, 30). Data on the relationship of some CCs to clinical symptoms are currently rather scarce, and research on this subject is quite limited. For example, CC30 is known to be associated with endocarditis (31), while no clear-cut correlations have been observed in AD. The only known example in the literature is the correlation between more frequent CC1 detection in AD patients with FLG mutations (32).

The question remains whether CC7 is somehow better adapted to colonizing atopic skin than other clones much less frequently detected in this study. Answers to these questions are being sought by studying virulence factors whose genetic determinants are located on mobile genetic elements. An example of such factors is SAgs. Based on our genetic analyses of the 4 classical enterotoxins and TSST-1, we showed that the gene distribution was heterogeneous among the different *spa* or MLST-CC types but also clonal. We were able to observe some trends that seem interesting. More than 60% of the *sec* genes, which were the most frequently detected SA-encoding genes in our population, were present mainly in CC45, CC7, and CC12. The high frequency of the *sec* gene was associated with many isolates belonging to CC7, as it was the most abundant clone in our population. In turn, the clones characterized by a high frequency of *sec* gene detection were dominated by CC12 (100% of isolates were positive for *sec*), CC72 (100%), and CC45 (75%). In the case of CC7, the frequency of gene detection was 37% and was close to CC15 (38.5%) and CC97 (33%). The same phenomenon was observed for *spa* types, i.e., 54% of detected *sec* genes were attributed to only 5 *spa* types (Table 6).

In the population we studied, we identified 5 MRSA isolates. They belong to CC8 (2), CC59 (2), and CC5 (1), the most popular genetic backgrounds of MRSA clones circulating in Polish hospitals in the first decade of this century (33). Such a low number of MRSA isolates seems to be in accordance with the numbers published from other European countries, 3% and 7.9% (4, 34), and in contrast to some observations from outside Europe, 30.8% (35).

A meta-analysis of 95 studies indicated that the rate of toxin-producing S. aureus on the lesional skin fluctuated between 31.5 and 80% and was higher than that on healthy control skin (36). Nevertheless, the percentage distribution of particular toxin-encoding genes varied depending on the geographical population studied. In the case of the selected toxin-encoding genes examined in this study, we did not observe a random distribution but rather an association with specific CCs. The SEs we examined were assembled on mobile genetic elements (i.e., phages or pathogenicity islands) and can therefore be transferred to other bacterial cells horizontally and vertically. However, data from the literature show that the SAg distribution is rather vertical (37). Like previously published data, we observed that *tsst-1* was associated with CC30 (37). The *sec* gene, which predominated in our studied population, was detected mainly in CC45 and CC7 isolates. All these results suggested that horizontal transfer of these genetic elements is not common. In contrast, *sea*, which is located on phage Φ3, was distributed much more broadly, and detected mostly in CC30 (5 of 7 isolates), CC97 (8 of 15 isolates), CC7 (16 of 35 isolates), and CC45 (8 of 25 isolates), but occasionally also in CC15, CC12, and CC1. The SAg-encoding gene *sed* (plasmid origin) was mainly detected in CC22 and in the same clones as *sea* (CC7, CC15, CC45, CC12, CC8, and CC30).

Some authors suggest that PVL-producing S. aureus strains play a key role in relapse of dermatitis in AD patients (38). According to recent reports, the *lukS/lukF*-*PV* genes are more common in isolates from pediatric patients and are detected in both MRSA and MSSA strains (39–41). Additionally, PVL-producing S. aureus corresponds to the severity of AD symptoms, as it was more often detected in children with moderate and severe SCORing Atopic Dermatitis (SCORAD) values (39). In our study, *lukS/lukF*-*PV* genes were found in 12.9% of isolates (18 of 139) and were more common in children (14 of 18 isolates, 77.7%). Our findings correspond with other observations regarding low *lukS/lukF*-*PV* detection rates (4, 40). According to our results, in the Polish S. aureus population from AD patients, *lukS/lukF*-*PV* genes were most common in CC45, CC22, CC7, and CC59. It is worth noting that the S. aureus 079N strain (ST1027) with the newly identified *spa* type also carried this gene. On the other hand, genes coding for PVL dominated in CC30 in the Brazilian AD population, and in CC5, CC8 and CC30 strains in the Canadian AD population (40, 41). The above data indicate no correlation between the clonal complex and the presence of the *lukS/F*-*PV* genes among S. aureus from AD patients.

Our results show that a large percentage (40%) of the tested isolates among skin/nose pairs are nonrelated or distinguishable strains, i.e., belonging to different *spa* types. This result may be partly because a high percentage of nonrelated pairs of isolates originated from children (48%). Unlike the second group of skin/nose paired strains, which were genetically indistinguishable and belonged to the same *spa* types, the percentage of isolates from children was only 28.5%. Such a high frequency of occurrence of various isolates in the same AD patient has not been previously observed and reported in the literature. In studies described in 2021 by Masiuk et al. (42), only two of 16 S. aureus from AD patients had a different genotype when nose and skin isolates were compared. Similarly, most AD patients have been found to be persistent S. aureus carriers with identical *spa* types in the nose and skin (43). The authors of previously published studies have also noted a high percentage of related skin and nasal isolates when they originated from one patient (4, 44, 45).

Due to the frequent use of topical and systemic antibiotics, AD patients are exposed to more antibiotics, which may select for more antibiotic resistant organisms (46). Our studies showed a relatively low percentage of resistance to local staphylococcal mupirocin (10%) and fusidic acid (6.5%). Such a low percentage may be because mupirocin is more often used for the decolonization of MRSA (20), which accounted for a small part of the studied population of isolates. For fusidic acid, a low rate of resistance may be due to short-term treatment with antibiotics. In the treatment of skin infections, a short period of using fusidic acid is recommended to avoid the development of resistance (47). On the other hand, our studies showed a high rate of erythromycin resistance (39%). The use of erythromycin may be limited due to the increasing resistance to macrolides (48). According to literature reports, macrolides are a first-line alternative to penicillin antimicrobials for penicillin allergic AD patients (28). Growing resistance to these groups of antibiotics may limit therapeutic options.

Antimicrobial photoinactivation is a viable option for treating S. aureus. In our studies, we showed that S. aureus is vulnerable to aPDI with the use of RB in combination with visible light (λ_max_ = 515 nm). Practically all the AD strains studied were effectively photoinactivated. More importantly, the efficacy of the photoinactivation process was independent of the presence and production of SAgs. This result means that RB-mediated aPDI applied against toxigenic strains is equally effective as it is against nontoxigenic strains. This finding agrees with studies published previously regarding SE-producing strains but with the use of different photoactive compounds, namely, porphyrin derivatives (49). RB is a commonly available compound that has been shown to be safe in animal studies on rabbit corneas (50). RB-based aPDI demonstrated efficacy in decreasing the parasitic load (Acanthamoeba castellanii) and clinical severity of actinic keratitis (51). Moreover, its photodynamic efficacy was positively verified in a mouse skin abrasion model of Pseudomonas aeruginosa infection (52, 53). Additionally, RB derivatives have been tested in a mouse wound healing model and shown to promote antistaphylococcal effects in a complex tissue model (54). Research on various RB delivery systems is also being conducted to modulate the action of RB (55). PH-10 is a topical RB hydrogel formulation that selectively delivers RB to the epithelial tissue and is currently being tested in clinical trials (NCT00941278, NCT1247818, NCT12322086, and NCT00690807) for the treatment of psoriasis and atopic dermatitis with encouraging results, although the mechanisms of action of RB in these diseases are not yet fully understood. RB can be used in the clinic as a topical drug, both as a light-free and light-activated compound. In the latter case, appropriate clinical studies are needed to verify the safety of RB as a potentially phototoxic compound. To date, there are no publicly available data from the only clinical trial on the use of a photodynamic method based on PH-10 in combination with green light (544 nm) in patients with psoriasis. Previous research together with our *in vitro* results (Fig. 3 and 4) indicate that RB can be a good candidate for antistaphylococcal treatment. It must be emphasized that RB is active not only against S. aureus but also against other microbial species *in vitro*, e.g., Bacillus subtilis, Staphylococcus epidermidis, Enterococcus faecalis, Streptococcus salivarius, *Streptococcus pneumoniae*, Mycobacterium smegmatis, Mycobacterium avium, Pseudomonas aeruginosa, *Salmonella Thyphimurium* (56). This means that the potential application of RB is much broader than in the presented manuscript, where only S. aureus was used as a model organism.

Information on the response of skin cells to light and rose bengal is of interest to scientists involved in the application of photodynamic methods, but data on keratinocytes are quite sparse in this regard. Extensive studies have been performed on the HeLa cell line exposed to light-activated RB (530 nm), showing that apoptosis and autophagy are the crucial events responsible for cell death, while necrosis is negligible (57, 58). Other cancer cell lines have also been studied (e.g., HL60, MCF-7, A431), while studies on the mechanisms of cell death in healthy skin cells have not yet been published. Green light, *per se*, is generally not considered toxic to keratinocytes. However, as mentioned above, when combined with certain photosensitizing agents, such as rose bengal, green light can induce cell death in keratinocytes due to oxidative damage that may ultimately lead to cell death. It should be emphasized, however, that our proposed RB-based photodynamic treatment had no mutagenic effect either in combination with green light or when green light was used alone (Fig. S1).

Whether aPDI approach would improve the functioning of atopic skin, prevent periodic worsening of the disease, or reduce its frequency requires further studies in murine models of atopy and in humans over the long term. Nevertheless, in the case of chronic diseases, such as AD, periodic, repeatable treatment is undoubtedly required, which raises the risk of selecting for resistance when using classic antibiotics. Such risk is minimized in the case of aPDI due to its completely different mechanism of action (59). In this respect, aPDI may constitute a viable alternative to antibiotics.

## MATERIALS AND METHODS

### Bacterial strains and growth conditions.

Three groups of S. aureus strains were used in the study. The first group comprised 139 clinical strains from patients with AD, isolated from the skin and nose from each patient in years 2014 and 2015 (Department of Dermatology, Venerology, and Allergology, Medical University of Gdańsk). The second group was 39 nonatopic isolates from the collection of S. aureus strains in the Laboratory of Photobiology and Molecular Diagnostics (IFB). The last group included 14 S. aureus reference strains, which have been characterized according to toxin production and genetic background (National Medicines Institute, Warsaw, Poland).

Bacterial cultures were grown aerobically in a nutrient trypticase soy broth (TSB, bioMérieux; France) at 37°C with shaking (150 rpm) or on trypticase soy agar (TSA, bioMérieux; France).

### Antimicrobial susceptibility testing.

The MIC was determined using the broth microdilution method according to the CLSI guidelines (ref. CLSI 2018). Assays for daptomycin were performed in medium supplemented with Ca^2+^ (50 mg/L). Polypropylene 96-well plates with bacteria at an initial inoculum of 5 × 10^5^ CFU/mL and exposed to the tested antibiotics were incubated for 18 h at 37°C. The following antibiotics were tested: ciprofloxacin (CIP), daptomycin (DAP), erythromycin (E), fusidic acid (FA), linezolid (LZD), mupirocin (MUP), tetracycline (TET), and vancomycin (Va). The experiments were performed in triplicate. The MIC values were categorized as resistant (R), intermediate (I), or susceptible (S) according to the EUCAST breakpoints for MIC interpretation (60). For mupirocin, MIC breakpoints were defined as susceptible, ≤4 mg/L; intermediate, 8 to 256 mg/L; and resistant ≥512 mg/L (61).

Methicillin resistance was assessed by culturing S. aureus isolates on Mueller-Hinton (4% NaCl) agar plates with oxacillin (Graso Biotech, Poland). Isolates were also screened by PCR for the presence of the *mec*A and *mec*C genes, as described earlier (62, 63).

### Isolation of genomic DNA.

Bacterial DNA was isolated from 1.5 mL of the overnight culture. Cells were pelleted by centrifugation at 13,400 rcf for 5 min (centrifuge 5415 D, Eppendorf). The pellet was suspended in 100 μL of lysis buffer (20 mM Tris-HCl pH 8.0, 2 mM EDTA pH 8.0, 1.2% Triton X-100) and 10 μL of lysostaphin (0.4 U/μL, A&A Biotechnology, Poland). For a more efficient isolation procedure, 120 mg of glass beads was added (Glasperlen, 0.10 to 0.11 mm, Sartorius StedimBiotech GmbH, Goettingen, Germany). The mixture, after mixing well by vortexing for 1 min, was incubated at 37°C for 40 min. During the incubation process, every 10 to 15 min, the mixture was briefly vortexed (10 to 15 s). The remaining DNA isolation steps were carried out using a genomic minikit (A&A Biotechnology, Poland) according to the manufacturer’s instructions with the addition of an RNA digestion step (RNase, 10 mg/mL, A&A Biotechnology, Poland). The DNA concentrations were measured using a NanoDrop ND-1000 (Thermo Scientific, USA).

### Detection of staphylococcal virulence genes.

The presence of genes encoding the five most common S. aureus toxins, enterotoxins A (*sea*), B (*seb*), C (*sec*), and D (*sed*) and toxic shock syndrome toxin-1 (*tsst*-*1*), was detected by PCR using specific primers based on Salgado-Pabón et al. (64) (Table 7). The total volume of 25 μL of reaction mixture contained 1× DreamTaq Buffer (including 2 mM MgCl_2_; Thermo Scientific, USA), 200 μM dNTPs (Thermo Scientific, USA), 0.5 pmol of each primer (TIB MOLBIOL Syntheselabor GmbH; Berlin, Germany), 0.5 U of DreamTaq polymerase (Thermo Scientific, USA) and 20 to 30 ng of template DNA. The presence of the *luk*S-*PV* and *lukF*-*PV* genes encoding Panton-Valentine leukocidin (PVL) was determined according to Lina et al. (65).

**TABLE 7:** Sequences of primers used in the study

Gene	Primers	Sequence of primers (5′–3′)	Product size (bp)
*sea*	*sea*-F *sea*-R	gAT TCA CAA Agg ATA TTg TTg ATA AAT AT gTC CTT gAg CAC CAA ATA AAT C	400
*seb*	*seb*-F *seb*-R	gTA TgA TgA TAA TCA TgT ATC AgC AA CgT AAg ATA AAC TTC AAT CTT CAC AT	625
*sec*	*sec*-F *sec*-R	gAg TCA ACC AgA CCC TAT gCC CgC CTg gTg CAg gCA TC	650
*sed*	*sed*-F *sed*-R	gCA TTA CTC TTT TTT ACT AgT TTg gTA CCT TgC TTg TgC ATC TAA TTC	530
*tsst-1*	*tsst-1*-F *tsst-1*-R	gAA ATT TTT CAT CgT AAg CCC TTT gTT g TTC ATC AAT ATT TAT Agg Tgg TTT TTC A	655

### Detection of staphylococcal toxin proteins: Western blotting.

For preparation of bacterial protein lysates, the overnight bacterial cultures were reinoculated at a ratio of 1:100 in 5 mL of sterile TSB medium and incubated until the late log phase of growth was reached (6 to 7 h; OD_600_ = 1.5 to 1.6). A total of 200 μL of each sample were suspended in a ratio of 1:1 in 2× Laemmli sample buffer (with the addition of β-mercaptoethanol; BioRad, USA) and incubated for 5 min in a thermoblock heated to 95°C. After incubation, the samples were briefly centrifuged (1 min, 16,100 rcf). The prepared bacterial protein lysates were stored at −20°C until further analysis.

The total protein concentration in protein lysates was measured. Due to the presence of reducing agent (β-mercaptoethanol), detergent (SDS) and dye (bromophenol blue) in Laemmli buffer, the total protein concentration in protein lysates was measured using an RD DC protein assay kit (BioRad, USA). A γ-globulin standard (BioRad, USA) was used as the protein standard at three different concentrations: 0.37 mg/mL, 0.74 mg/mL, and 1.48 mg/mL. The amount of total protein was measured spectrophotometrically using a UV-VIS SPECORD 2000 PLUS spectrophotometer (Analytik Jena, Germany) at a wavelength of 750 nm. The absorbance values of the tested samples were compared with the pattern of the curve of the γ-globulin protein standard, yielding the values of total protein concentration in the prepared bacterial lysates.

For sodium dodecyl sulfate-polyacrylamide gel electrophoresis (SDS-PAGE), 5 μL of a protein marker (PageRuler prestained protein ladder, 10 to 180 kDa; Thermo Fisher Scientific, USA), 5 μL of the protein standard of the tested SAgs at a concentration of 25 μg/mL (Toxin Technology Inc., USA), and 10 μg of protein lysates of the tested samples were applied to the wells of a polyacrylamide gel. SDS-PAGE was carried out in 1× concentrated SDS-PAGE buffer in a Mini-PROTEAN Tetra Cell vertical electrophoresis apparatus (BioRad, USA) at a voltage of 180 V for 60 min.

### Electrotransfer and immunodetection.

Wet transfer was performed using a polyvinylidene fluoride (PVDF) membrane (BioRad, USA). A cassette with sandwiches was placed in a Mini-PROTEAN Tetra Cell apparatus (BioRad, USA) along with a cooling cartridge. Membrane transfer was performed on ice in 1× concentrated transfer buffer at 100 V for 60 min. The PVDF membrane was blocked for 30 min in 30 mL of Tris-buffered saline + Tween (TBST) solution with 1% skim milk and shaken on a rocking shaker at room temperature. The membrane was washed twice with 30 mL of TBS-Tween, incubated with a 1:10,000 dilution of the primary antibody (antistaphylococcal enterotoxin IgG; Toxin Technology Inc., USA) in 30 mL of TBST solution with 1% skim milk overnight at 4°C with gentle shaking. The next day, three 5-min washes were performed with 30 mL of TBST with gentle shaking on a rocking shaker. Horseradish peroxidase (HRP)-labeled secondary antibodies at a ratio of 1:10,000 (peroxidase-conjugated AffiniPure Alpaca antirabbit IgG; Jackson ImmunoResearch Laboratories Inc., USA) in 30 mL of TBST solution with 1% skim milk were added and incubated for 30 min on a rocking shaker at room temperature. After this time, three 5-min washes were performed with 30 mL of TBST. Finally, the membrane was washed twice with 30 mL of Tris-buffered saline (TBS). The membrane was covered with Clarity Max ECL substrate (BioRad, USA), and the chemiluminescence signal was read on a ChemiDoc XRS + documentation system (BioRad, USA). The obtained image was analyzed in Image Lab (version 6.0, BioRad, USA). The obtained band intensities for individual samples were analyzed against the standard curve of the enterotoxin protein standard. The amount of protein of a given isolate was tested in three independent experiments (from three independent cell cultures).

### Antimicrobial photodynamic inactivation (aPDI).

**(i) Chemicals.** Rose bengal (RB) (4,5,6,7-tetrachloro-2′,4′,5′,7′-tetraiodofluorescein disodium salt; Sigma-Aldrich, Germany) was used in this study as a light-activated molecule (photosensitizer). A 1 mM RB stock solution was prepared in sterile double-distilled water and stored at 4°C for up to a month. Before use, the stock solution was diluted in sterile double-distilled water to the appropriate concentration.

**(ii) Light source.** The illumination process was carried out using an LED lamp (SecureMedia, Poland) emitting green light (λ_max_ = 515 nm) (66). The output power was 35 mW/cm^2^. The bacterial cultures were irradiated with a total dose of light of 10, 20, or 40 J/cm^2^ (duration of the illumination process of 333, 666, or 1332 s, respectively).

**(iii) Phototreatment experiment.** Bacterial cultures were grown overnight (18 ± 2 h) at 37°C in nutrient TSB (bioMérieux, France) and then diluted with fresh medium to a density of 0.5 McFarland units (approximately 10^7^ CFU/mL). Diluted S. aureus cultures (200 μL) were incubated in the dark at 37°C with shaking (150 rpm) for 10 min, either with or without the photosensitizer. The final concentrations of the photosensitizer were as follows: 0.5 μM and 1 μM. After the incubation process, 100 μL of each culture was transferred into a 96-well plate and irradiated with different light doses of 10, 20, and 40 J/cm^2^. The remaining 100 μL of S. aureus cultures incubated with and without the photosensitizer in the dark during the illumination process served as controls. After the irradiation time, 10 μL of each sample was taken to perform 10-fold serial dilutions (from 10^−1^ to 10^−4^) in sterile PBS. Then, 10 μL of each dilution were streaked horizontally onto TSA plates (bioMérieux, France) and incubated for 24 h at 37°C. After overnight incubation, the bacterial colonies were counted, and the results were statistically analyzed. Survival fractions were expressed as ratios of CFU of treated bacteria (with light and photosensitizer) to CFU of untreated bacteria. Each experiment was performed in triplicate (from three independent cultures of each isolate).

**(iv) Spa typing.** Amplification of the variable-number tandem repeat (VNTR) region of the *spa* gene in S. aureus strains from AD patients was conducted by using PCR with specific primers based on Aires-de-Sousa et al. (67) (Table 8). The amplification method was performed as follows: a total volume of 50 μL of the reaction mixture contained 1× DreamTaq Buffer (including 2 mM MgCl_2_; Thermo Scientific, USA), 100 μM dNTPs (Thermo Scientific, USA), 1 pmol of each primer (Eurofins Genomics, Ebersberg, Germany), 0.05 U of DreamTaq polymerase (Thermo Scientific, USA), and 1 μL of template DNA.

**TABLE 8:** Sequences of primers used in the study^*a*^

Gene	Primers	Sequences of primers (5′–3′)	Product size (bp)
*spa*	*spa*-1113F	TAA AGA CGA TCC TTC GGT GAG	100–422
	*spa*-1514R	CAG CAG TAG TGC CGT TTG CTT	

a *spa* types were determined by using the Ridom StaphType software v.2.1.1 (Ridom GmbH; Münster, Germany) (70).

Amplification was carried out in a Gene Amp PCR System 9700 (Applied Biosystems) using the following steps: initial denaturation (94°C, 5 min) followed by 30 cycles of denaturation at 94°C for 30 s, annealing of primers at 63°C for 30 s and extension at 72°C for 45 s. The reaction was terminated with extension at 72°C for 3 min.

PCR products were analyzed by electrophoresis through a 1% agarose gel in 0.5× TBE buffer (diluted from 10× TBE buffer, Thermo Scientific, USA), stained with a safe replacement for ethidium bromide (Simply Safe; EURx, Poland) and visualized under UV illumination using a ChemiDoc imaging systems instrument (BioRad, USA). The PCR products were sequenced by Eurofins Genomics AT GmbH (Austria) using the primers used in the PCR. Sequencing of both complementary strands of the PCR product was performed.

A minimum spanning tree (MST) was created based on *spa* type profiles using Ridom SeqSphere+ software version 8.3.1 (Ridom GmbH; Münster, Germany) with default parameters enabled during comparison: MST Clusters cost distance - 5 and exclude *spa* types shorter than 5 repeats, options turned on in the SeqSphere+ software during comparison (68).

Multilocus sequence typing (MLST) was performed based on Enright et al. (69) for 13 MSSA isolates characterized by a new *spa* type or *spa* type for which sequence type (ST) could not be predicted. Allele numbers and STs were assigned through the S. aureus MLST database (http://pubmlst.org/organisms/staphylococcus-aureus). For remaining isolates, ST and MLST-clonal complex (MLST-CC) were inferred from the *spa* type in the Ridom SpaServer (http://spaserver.ridom.de/) and from the literature (70).

### Photo- and cytotoxicity assays based on MTT.

The human keratinocyte cell line (HaCaT, CLS 300493) was cultured in Dulbecco’s modified Eagle’s medium (DMEM) supplemented with 4.5 g/L glucose, 1 mM sodium pyruvate, 10% fetal bovine serum, 100 U/mL penicillin, 100 μg/mL streptomycin, 2 mM l-glutamine, and 1 mM nonessential amino acids (all reagents from Gibco; Thermo Fisher Scientific, USA). The cultures were grown in a humidified atmosphere containing 5% CO_2_ at 37°C.

Cells (1 × 10^4^) were seeded in a 96-well plate and allowed to adhere overnight. RB at the final concentration of 0 to10 μM was examined. The cells were incubated with RB for 15 min at 37°C in the dark, washed once with Dulbecco’s phosphate-buffered saline (DPBS; Sigma-Aldrich, Germany), and dissolved in 100 μL of DMEM (Gibco; Thermo Fisher Scientific, USA). Next, irradiation was performed using green light (λ_max_ = 515 nm, 40 J/cm^2^, 35 mW/cm^2^, 1332 s). Cell survival was determined after 24 h of incubation by an MTT assay using MTT reagent (3-[4,5-dimethylthiazol-2-yl]-2,5-diphenyltetrazolium bromide, Sigma–Aldrich, Germany). Next, 10 μL of MTT reagent (12 mM stock solution) were added to each well, and then the cells were incubated for 4 h at 37°C. Then, cells were lysed in 100 μL of dimethyl sulfoxide (DMSO; Sigma-Aldrich, Germany), and the absorbance of the formazan solution was measured at 550 nm using a plate reader (Victor 1420 Multilabel Plate Reader, Perkin-Elmer). All experiments were carried out in three biological replicates for each condition (cells treated with light and untreated cells stored in the dark). Cell viability (expressed as a percentage) is presented as the mean of the three independent biological replicates with reference to the untreated cells.

### Statistical analysis.

All statistical calculations were performed using the StatSoft Inc. statistical package (2014) STATISTICA (data analysis software system, version 12.0, www.statsoft.com) and an Excel spreadsheet. Quantitative variables were characterized by the arithmetic mean, standard deviation, median, minimum, and maximum value (range) and 95% confidence interval (CI). On the other hand, the qualitative variables are presented by means of the count and percentage values (percentage). The significance of difference between two groups was tested by means of Student's *t* test or the Mann-Whitney U test. Chi-square tests of independence were used for qualitative variables (using Yates correction for cell counts below 10, checking Cochran conditions, Fisher's exact test, respectively). In all calculations, *P* = 0.05 was adopted as the level of significance.

### Conclusions.

The S. aureus population colonizing the skin of AD patients is heterogeneous and largely reflects the distribution of S. aureus isolates circulating in the general population. Noteworthy, however, is the 4-fold increased number of isolates belonging to CC7 that we identified in the Polish population. The question of whether CC7 is particularly predisposed to colonization of atopic skin remains open.

The most frequently detected superantigen genes among our subjects were *sec* and *sea*; we also observed a difference in the distribution of genes encoding the tested superantigens between the group of adults and children. Regardless of the presented pattern of genes encoding enterotoxins, S. aureus is highly sensitive to phototreatment under conditions that are safe for human keratinocytes. The heterogeneous S. aureus population is effectively inactivated *in vitro* by the action of aPDI, which provides a reasonable basis for validating aPDI as an antimicrobial method in more complex *in vivo* models.
