# Supplementary material for: Staphylococcus aureus from Atopic Dermatitis Patients: Its Genetic Structure and Susceptibility to Phototreatment
Source: Microbiol Spectr. 2023 May 4;11(3):e04598-22. doi: 10.1128/spectrum.04598-22 (PMC10269521; doi:10.1128/spectrum.04598-22)
Supplement: Supplemental file 1 — Supplemental material. Download spectrum.04598-22-s0001.pdf, PDF file, 1 MB [file spectrum.04598-22-s0001.pdf]

Supplemental Table 1

Table S1 Distribution of staphylococcal toxin genes in non-atopic patients.

Strain	Isolate no.	Patient	Site of isolation	MRSA/MSSA	*sea*	*seb*	*sec*	*sed*	*tst*
**1**	**95**	Adult	Skin	MSSA	***sea***	-	-	-	-
**2**	**118**	Child	Nose	MSSA	-	***seb***	-	-	-
**3**	**119**	Child	Throat	MSSA	-	-	-	-	-
**4**	**138**	Child	Throat	MSSA	-	-	-	-	-
**5**	**140**	Child	Nose	MSSA	-	***seb***	-	-	-
**6**	**269**	Child	Ear	MSSA	-	-	-	-	-
**7**	**591**	Adult	Nose	MSSA	-	-	-	-	-
**8**	**598**	Child	Throat	MSSA	-	***seb***	-	-	-
**9**	**970**	Child	Throat	MSSA	-	-	***sec***	-	-
**10**	**1327**	Child	Nose	MSSA	-	-	***sec***	-	-
**11**	**1385**	Child	Ear	MSSA	-	-	-	-	-
**12**	**1457**	Adult	Bronchial tree	MSSA	***sea***	-	-	-	-
**13**	**1917**	Child	Umbilicus	MSSA	-	-	***sec***	-	-
**14**	**2086**	Adult	Nose	MSSA	-	-	***sec***	-	-
**15**	**2469**	Child	Anus	MSSA	-	-	-	-	-
**16**	**3076**	Child	Skin	MSSA	-	-	-	-	-
**17**	**3097**	Child	Throat	MSSA	-	-	-	-	-
**18**	**3098**	Child	Ear	MSSA	-	-	-	-	-
**19**	**3149**	Child	Ear	MSSA	-	-	-	-	-
**20**	**3840**	Child	Ear	MSSA	-	-	-	***sed***	-
**21**	**4597**	Child	Nose	MSSA	-	-	-	-	-
**22**	**4759**	Child	Ear	MSSA	-	-	-	-	-
**23**	**6558**	Adult	Bronchial tres	MSSA	***sea***	-	-	-	-
**24**	**10221**	Child	Throat	MSSA	***sea***	-	-	-	-
**25**	**13289**	Child	Skin	MSSA	***sea***	-	-	-	-
**26**	**15587**	Child	Nose	MSSA	***sea***	-	-	-	-
**27**	**1159/S**	Adult	Bronchial tree	MSSA	-	-	-	-	-
**28**	**1162/0**	Adult	Bronchial tree	MSSA	-	-	-	***sed***	-
**29**	**1289/2**	Child	Umbilicus	MSSA	-	***seb***	-	-	-
**30**	**1611/S**	Adult	Nose	MSSA	-	-	-	-	-
**31**	**1964/S**	Adult	Nose	MSSA	-	-	-	-	-
**32**	**2028/S**	Adult	Nose	MSSA	-	-	-	-	-
**33**	**345/0/7**	Adult	Nose	MSSA	-	-	-	-	-
**34**	**491/e**	Child	Nose	MSSA	-	-	-	-	-
**35**	**529/e**	Adult	Nose	MSSA	-	-	-	-	***tst***
**36**	**564/E**	Adult	Nose	MSSA	-	-	-	-	-
**37**	**5718/2**	Adult	Bronchial tree	MSSA	-	-	-	***sed***	-
**38**	**80/0**	Adult	Bronchial tree	MSSA	***sea***	-	-	-	-
**39**	**990/P/7**	Adult	Throat	MSSA	-	-	-	-	-

MSSA, methicillin sensitive *Staphylococcus aureus*; *sea*, staphylococcal enterotoxin A; *seb*, staphylococcal enterotoxin B; *sec*, staphylococcal enterotoxin C; *sed*, staphylococcal enterotoxin D; *tst*, toxic shock syndrome toxin-1

Suplemental Figure 1

A)

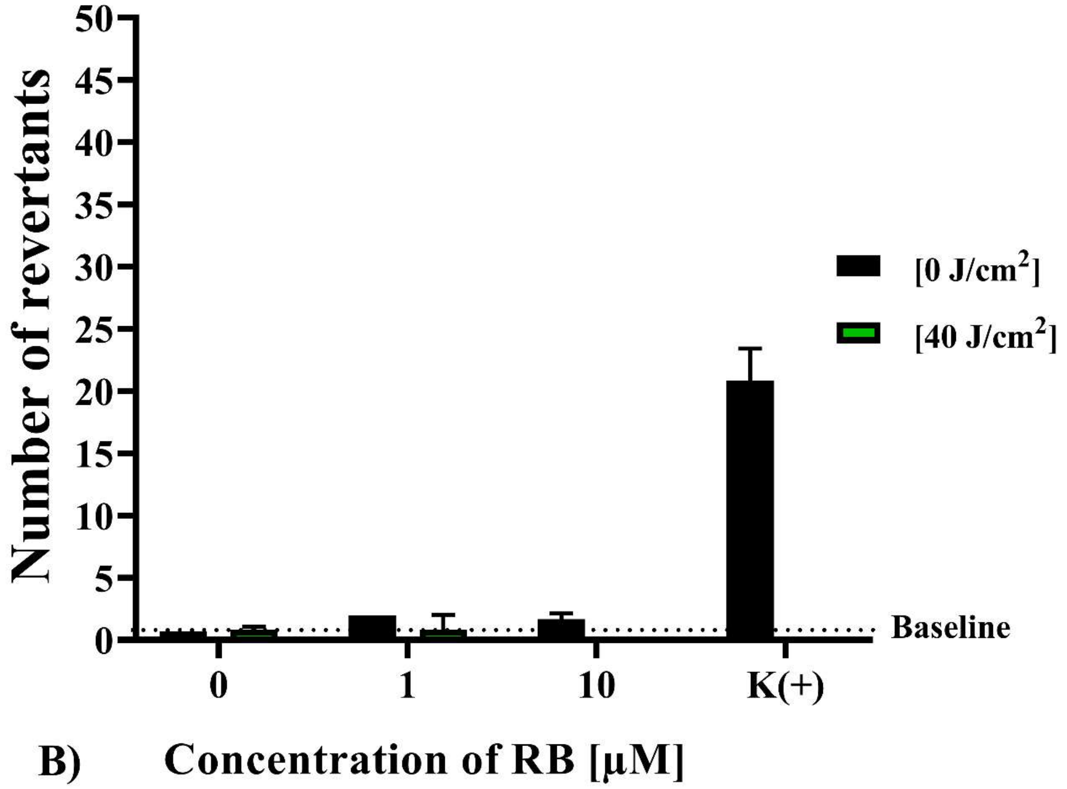

B)

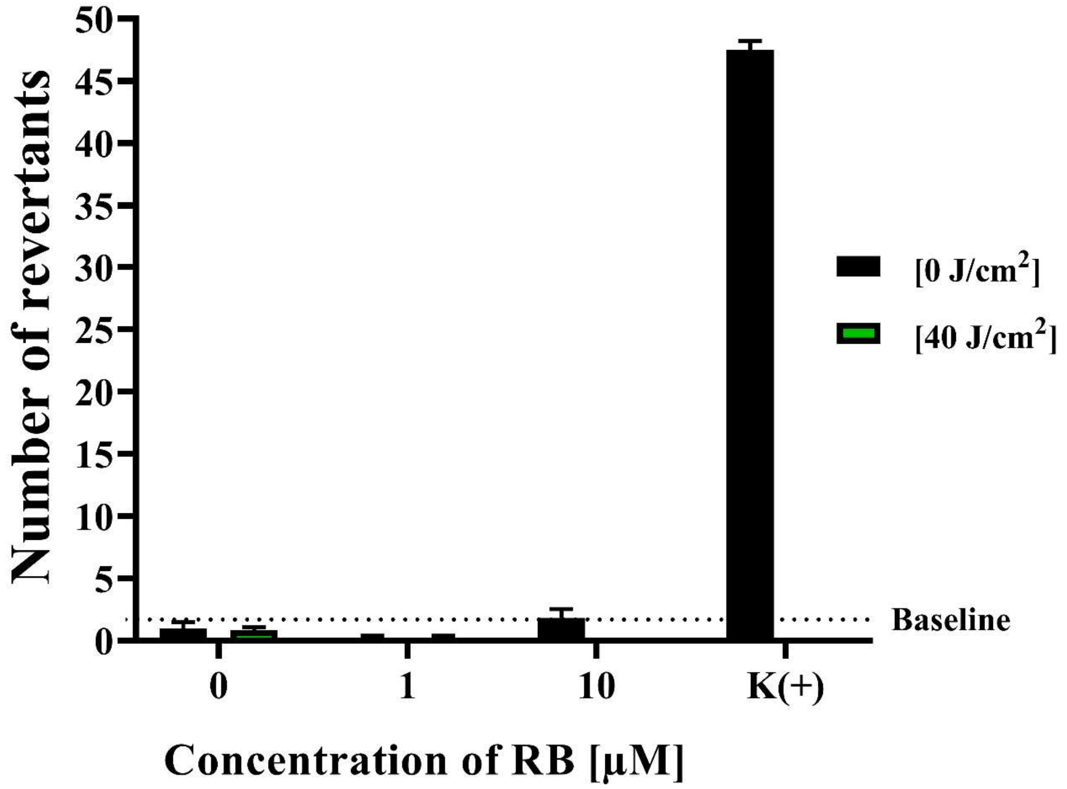

Fig. S1. Photoinactivation mutagenicity assay based on rose bengal (RB) and green light. Mutant strains of *E. coli uvrA* (A) and *Salmonella Typhimurium TA98* (B) were treated with RB in the dark [0 J/cm^2], with light-activated RB [40 J/cm^2], or with light alone [40 J/cm^2] in an Ames Penta-2 apparatus (Xenometrix, Allschwil, Switzerland). The measure of mutagenicity was the number of revertants counted for each treatment compared to two types of controls: negative - untreated cells (0 µM RB, 0 J/cm^2) and positive - chemically induced revertants using 4-NQO for *E. coli uvrA* and 2-NF for *S. typhimurium TA98*. The experiment was performed for three independent biological replicates with three technical replicates. The values obtained are mean revertant counts with SD.

Methodology:

Prokaryotic mutagenicity assay was performed using a commercial kit Ames Penta 2 (Xenometrix, Allschwil, Switzerland). All steps were performed according to the manufacturer's protocol. Two independent biological cultures of each tested strain *Escherichia coli uvrA* or *Salmonella Typhimurium TA98* were established the day before the experiment. After incubation at 37 °C for 14 hours with shaking, the cultures were diluted in exposure medium and treated with rose bengal at a concentration of 0-10 µM. Cultures were incubated in the dark for 10 min and then either illuminated with green light (40 J/cm^2) or left in the dark (0 J/cm^2). As positive controls, cultures were treated with mutagenic chemical agents such as 4-nitroquinoline N-oxide (4-NQO) for *E. coli uvrA* or 2-nitrofluorene (2-NF) for *Salmonella Typhimurium TA98*. As a negative control, cells were incubated without a compound or light exposure. All samples were incubated for a total of 90 minutes after addition of the mutagen or RB-mediated aPDI treatment. Indicator medium was then added to all treatments, and 50 µl of each sample was distributed to the 384-well plates. All microtiter plates were incubated at 37 °C for 48 hours. Then, the number of revertants was counted after each treatment. The occurrence of revertants was detected by the color change of the indicator medium from purple to yellow. This experiment was performed with two independent biological experiments and three independent technical replicates.
